# Late Frontal Negativity Discriminates Outcomes and Intentions in Trust-Repayment Behavior

**DOI:** 10.3389/fpsyg.2020.532295

**Published:** 2020-11-25

**Authors:** Mauricio Aspé-Sánchez, Paola Mengotti, Raffaella Rumiati, Carlos Rodríguez-Sickert, John Ewer, Pablo Billeke

**Affiliations:** ^1^División de Neurociencia (NeuroCICS), Centro de Investigación en Complejidad Social, Facultad de Gobierno, Universidad del Desarrollo, Santiago, Chile; ^2^Centro de Investigación en Complejidad Social, Facultad de Gobierno, Universidad del Desarrollo, Santiago, Chile; ^3^Instituto de Neurociencia, Universidad de Valparaíso, Valparaíso, Chile; ^4^Neuroscience Area, Scuola Internazionale Superiore di Studi Avanzati, Trieste, Italy; ^5^Cognitive Neuroscience, Institute of Neuroscience and Medicine (INM-3), Jülich Research Centre, Jülich, Germany

**Keywords:** altruism, anterior cingulate cortex, dorsomedial prefrontal cortex, event-related potentials, positive and negative reciprocity, temporoparietal junction, theory of mind

## Abstract

Altruism (a costly action that benefits others) and reciprocity (the repayment of acts in kind) differ in that the former expresses preferences about the outcome of a social interaction, whereas the latter requires, in addition, ascribing intentions to others. Interestingly, an individual’s behavior and neurophysiological activity under outcome- versus intention-based interactions has not been compared directly using different endowments in the same subject and during the same session. Here, we used a mixed version of the Dictator and the Investment games, together with electroencephalography, to uncover a subject’s behavior and brain activity when challenged with endowments of different sizes in contexts that call for an altruistic (outcome-based) versus a reciprocal (intention-based) response. We found that subjects displayed positive or negative reciprocity (reciprocal responses greater or smaller than that for altruism, respectively) depending on the amount of trust they received. Furthermore, a subject’s late frontal negativity differed between conditions, predicting responses to trust in intentions-based trials. Finally, brain regions related with mentalizing and cognitive control were the cortical sources of this activity. Thus, our work disentangles the behavioral components present in the repayment of trust, and sheds light on the neural activity underlying the integration of outcomes and perceived intentions in human economic interactions.

## Introduction

Due to their pervasiveness and functional importance in economic and social life, relations of trust have become an important research topic in the social sciences during the last decades, from sociology (Coleman, 2000; Cook and Santana, 2020) to economics (Dasgupta, 1988) and political sciences (Putnam et al., 1994; Putnam, 2000) to psychology (Dunning and Fetchenhauer, 2011). Situations involving trust constitute a subclass of those involving risk, in which the risk the trustor (voluntarily) takes depends on the response from the trustee, the party in which trust is placed (Coleman, 2000).

The use of game theoretical experimental paradigms, which reproduce the features of situations involving trust, have produced a new wave of empirical research that aids in our understanding of the determinants of both trusting behavior (on the role of the trustor) and trustworthy behavior (on the side of the trustee). A well-known experimental paradigm that stylizes these situations is the Trust game (TG; Camerer, 2003). In a TG, the trustor faces a binary choice to either trust or distrust the trustee; and the trustee—if trusted—also faces a binary choice to either honor this trust (case in which both the trustor and the trustee see their situation improved relative to the distrust scenario), or to abuse this trust. If trust is placed by the trustor and abused by the trustee, the trustee obtains a higher payoff than when he honors this trust, but the trustor sees his position impoverished with respect to the *status quo* (i.e., distrust). Similarly, in the Investment game (IG; Berg et al., 1995), the trustor receives a monetary endowment and decides how much of this endowment will be “invested” in the trustee. This investment is usually first tripled before being sent to the trustee, who now decides—after observing the trustor’s allocation—the amount of money that she/he will send back to the trustor. Thus, the IG provides behavioral measures of trust—the level of investment by the trustor—and trustworthiness—the repayment by the trustee (Camerer, 2003). Experimental studies of trust games have recently been informed by the field of social neuroscience and its enquiries into the neurobiological correlates of behavior occurring during these games.

Whereas most studies focus on the behavioral and neurobiological correlates of trusting behavior (see, for instance, Kosfeld et al., 2005; Baumgartner et al., 2008, 2011), here we focus on the trustee’s response, and thus on the behavioral determinants of trustworthiness and its neurobiological correlates. In the IG, specifically, trusting behavior has been mostly associated with expectations about trustworthiness (Ashraf et al., 2006) and betrayal aversion (Camerer, 2003). On the other hand, trustworthy behavior has been mostly associated with other-regarding behaviors (Fehr, 2009). Other-regarding behavior may be related to the outcomes of the game or to the intentions inferred by the trustee from the trustor’s actions (McCabe et al., 2003). Outcome-based behavior could take the form of altruism—i.e., a costly unconditional act that benefits another individual (Wilson, 1975; Levine, 1998)—or inequity aversion (Bolton and Ockenfels, 2000; Fehr and Schmidt, 2001). It is important to stress that neither altruism nor inequity aversion depend on the intentions ascribed by the trustee to the trustor and, therefore, should not be influenced by the investment allocated by the trustor. Intention-based behavior, on the other hand, takes the form of reciprocity, the disposition to spend resources to reward favorable treatment or to sanction unfavorable treatment (Gouldner, 1960; Dufwenberg and Kirchsteiger, 2004).

When the trustor’s decision is binary, there is no ambiguity: Trust is a favorable act toward the trustee, and distrust is an unfavorable one. However, in the context of the IG, whether positive or negative reciprocity will influence the trustee’s decision will depend on whether she/he evaluates the level of investment as trust or distrust. Thus, the trustee might consider that only investments above a given threshold should be considered trust. If positive reciprocity (an intention-based behavior) is influencing the trustee’s behavior, the amount sent back by the trustee should be higher than the amount she/he would send if only altruism and inequity aversion (outcome-based behaviors) were influencing the trustee’s decision. In contrast, the amount sent back should be lower when negative reciprocity has been triggered.

Insights from neurocognitive studies have not disentangled intention- from outcome-based behaviors. Reports using the TG have shown that the mentalizing system—mainly the temporoparietal junction (TPJ; Frith and Frith, 1999; Decety and Lamm, 2007; Abu-Akel and Shamay-Tsoory, 2011)—is activated when a trustee reciprocates a trustor’s risky allocation (van den Bos et al., 2009). The TPJ has also been shown to be important in the control of selfish impulses (Hutcherson et al., 2015). The cognitive control system, which is crucial for the inhibition of selfishness, and for strategic and normative decision-making, may also be involved since the anterior cingulate (ACC; Delgado et al., 2005; van den Bos et al., 2009; Shenhav et al., 2013) and the dorsolateral prefrontal cortices (DLPFC; Baumgartner et al., 2011; Chang et al., 2011; Yamagishi et al., 2016) show increased activity when trustees repay trust with an amount that is smaller or greater than what they think the trustor expects to be repaid, respectively (Chang et al., 2011). However, both the mentalizing and the cognitive control networks are also involved under outcome-based conditions: The activity of the right TPJ correlates with how subjects value the outcomes of others (Hutcherson et al., 2015), whereas the connectivity between the ACC and the anterior insula predicts empathy-driven (outcomes-based) versus reciprocity-driven (intentions-based) altruism (Hein et al., 2016). Electrophysiologically, the frontomedial negativity (FMN), a family of event-related potential (ERP) deflections classically related to performance monitoring (Holroyd and Coles, 2002)—and whose source is the medial prefrontal cortex (Holroyd and Coles, 2002; Billeke et al., 2013; Cavanagh and Frank, 2014; Ullsperger et al., 2014)—is more pronounced when subjects receive an unfair versus a fair allocation (but specifically when a friend is playing the role of dictator; Wu et al., 2011). Importantly, to date no studies have used the greater temporal resolution of EEG to disentangle intention- and outcome-based neural activity.

In order to disentangle the influence of outcome-based preferences and intention-based preferences, and its neurobiological correlates, we used a mixed “Dictator/Investment” game (DIG), which uses, in the same session, both the IG and DG (based on Cox, 2004; see also Ashraf et al., 2006). This setup allowed us to compare a subject’s ERPs activity (i) when they received an amount from a human trustor (IG condition), versus (ii) when they received the same amount from a computer (DG condition). Under the IG condition, the decision to repay trust by the trustee was preceded by investment decisions by the trustor (an actual person) and thus could be the result not only of outcome-based behavior (altruism or inequity aversion), but also intention-based behavior (positive or negative reciprocity). In contrast, under the DG condition, the (same) endowment to be allocated by the trustee comes from a computer, and thus can only be associated with outcome-based behavior, because is independent from the intentions of the trustor. In addition, we compared the subsequent allocation that subjects made under both scenarios, and measured their EEG activity in the time windows where subjects were notified about the amount of money they were endowed. Our results showed that reciprocity actually consisted of positive and negative reciprocity, for high versus low amounts of received trust, respectively. At the neural level, a late frontomedial negativity was more prominent in outcome-based trials, whereas in intentions-based trials it predicted how subjects responded to trust. Interestingly, the medial and dorsolateral prefrontal cortices and the left temporoparietal junction were the sources of this neurophysiological activity. In summary, our work disentangles the different behavioral components underlying the repayment of trust, and implicates brain networks involved in mentalizing and cognitive control in the process of integrating outcomes and perceived intentions when humans engage in economic interactions.

## Methods

### Participants

Twenty right-handed undergraduate students (mean age = 21.2 years; s.d. = 2.07 years; min = 18 years; max = 25 years; 45% women) participated in the experiment. Participants were instructed to abstain from exercise, caffeine, and alcohol, starting the night before the sessions. Subjects with chronic diseases, mental disorders, medication, or those who abused drugs, were excluded. All subjects approved and signed a written informed consent form. They then read written and listened to verbal instructions explaining the task. Finally, they answered a 7 item questionnaire, to ensure that they had understood the logic of the game. All participants answered all questions correctly. The main experiments were carried out in the EEG Lab of the Neuroscience Area of the Scuola Internazionale Superiore di Studi Avanzati (SISSA), Trieste, Italy, while additional control experiments were carried out in Universidad de Valparaíso y Universidad del Desarrollo, Chile (see below). The experiments were performed according to the Declaration of Helsinki, and approved by the SISSA bioethics committee.

### Instruments

#### The DIG (Dictator-Investments Game)

**FIGURE 1:**
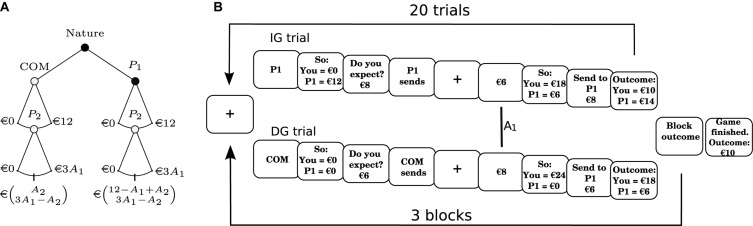
Experimental protocol. **(A)** Behavioral protocol. Schematic of the Dictator/Investment game (DIG) used here. Whether the subject faced a Dictator (DG; open node) or an Investment (IG; closed node) condition was decided randomly with a probability of 0.5 for each. Subjects always played as P_2_. The payoff matrix is at the bottom of the tree, with payoffs for P_1_ and P_2_ shown in the first and second row, respectively. **(B)** Flow of the game. Subjects played a total of 60 trials (in 3 blocks of 20 trials), consisting of 30 trials under the IG conditions (upper flow) and 30 trials under the DG conditions (lower flow). Trigger (vertical line) marks the moment when the subject was notified that an allocation (A_1_) had been made. COM: computer; P1: player 1 (the trustor); P2: player 2 (the trustee).

The DIG, which we introduce here, combines the classical IG (Berg et al., 1995) and DG (Forsythe et al., 1994) setups, based on the experiments made by Cox (2004) (see also Ashraf et al., 2006). We call P_1_ the trustor and P_2_ the trustee (as in a classical IG). In our experiment the focus is on P_2_. P_2_ subjects performed 60 trials of a recurrent-interactions DIG. In each trial, subjects played either an IG or a DG, which was decided using a pseudo-random distribution programmed so that each player played 30 IG trials and 30 DG trials. In both scenarios, the initial maximum amount of money available was equal to €12, and the exchange factor was equal to 3 (see Figure 1 and above). The DIG trials were divided into 3 blocks of 20 trials each. Participants were told that they would play with 20 different P_1_s, located in a different and dedicated room. Thus, they played three times with each P_1_ (once in each block), always in a random order, which prevented reputation-building motives. After they completed the task, they received an amount equal to the outcome of a random trial, plus a €10-base. All allocations were computer simulations sending a pseudo-random allocation drawn from a uniform distribution in the range of [0, 12] €.

#### IG Trials

In the IG trials of the DIG, P_2_ began with €0. P_1_s then sent an allocation A_1_ in the range [0, 12] €, which was multiplied by 3, and given to P_2_. P_2_ then decided how much of the amount they received (€3 × A_1_) to send back to the P_1_ they were paired with in this trial. The allocation made by P_2_ (A_2_) is considered to be an intentions-based allocation, given that the behavior of P_2_ would be influenced by the ascription of an intention to trust or cooperate, signaled by the allocation A_1_ of the P_1_ (see Berg et al., 1995; Cox, 2004; Ashraf et al., 2006).

#### DG Trials

In the DG trials of the DIG, P_2_ also began the game with €0. Unlike the IG, however, P_2_ was told that the amount A_1_ (also in the range of [0, 12] €) they received would be decided not by P_1_, but a computer (COM). This amount was then multiplied by 3 and given to P_2_. As in the IG, P_2_ then decided how much of the amount they received (3 × A1 €) to send to the P_1_ they were paired with in this trial. In this case, however, the allocation made by the P_2_ (A_2_) is considered to be an outcome-based allocation, given that their behavior would be influenced not by the ascription of a cooperative intention of P_1_ (because P_1_ did not decide how much COM sent to P_2_), but by concerns regarding the distribution of the total amount of money available.

### Experimental Procedure

#### Behavioral Task

Participants were seated in front of a computer monitor in a soundproof cabin. All electrical devices that could interfere with EEG acquisition were turned off. Stimuli were presented using the “Presentation” software^1^. In order to ensure that participants understood the dynamics of the game, they first played three practice trials of the DIG. Then, participants were left alone in the room and began to play the DIG (see Figure 1).

#### Flow of the Game

The flow of the DIG was as follows: In the first screen, participants faced a short (3 s) video of 20 individuals, some of them entering a room, to create the impression that the players would be interacting with humans. After that, a screen asked subjects to wait for the connection with the other participants to be established, then asked them to press a key when they wanted to begin the experiment. After that, a screen displayed a string saying that the program was searching for another human participant. Since subjects were in fact always playing with a COM, this screen was displayed for a random duration between 1 and 10 s, until a screen indicated that the P_1_ was ready, thereby giving the impression that the program was connecting via local area network to the room with the chosen P_1_s. When the program connected with the “human” partner, a screen displayed the ID of the partner for 2 s (for instance: “P_1_**:** FW253”), then displayed for 2 s a screen saying that the program was determining if the player would receive an amount from the COM or from the human partner. After that, a fixation cross was presented for a random duration between 3.5 and 5 s. After the fixation time, the screen indicated, for 3 s, if the participant was in a IG or in a DG trial, following which it displayed for 3.5 s the initial endowment of the players. Then, a screen asked participants how much they expected from P_1_ or COM. If the trial was an IG, a screen then displayed, for a random duration between 2 and 7 s (to make participants believe that another human was making a choice), a string notifying that P_1_ was sending an allocation. In a DG, the screen immediately displayed for 2 s a string saying that COM was sending a certain amount. After that, the fixation cross was again displayed for between 3.5 and 5 s, after which the amount that P_1_ or COM had sent was displayed for 3 s. If A_1_ > €0, a screen asked the participant to send an amount to P_1_, displaying a random amount between €0 and the total amount available for the participant (€3 × A_1_), waiting for the subject to use the buttons of the joystick to increase or decrease the amount. This method was used to avoid inducing participants to select a set amount (for instance, by always displaying €0 or the maximum available). A screen then showed the outcome of the trial, both for the participant and for P_1_, and asked the participant to press any key to continue to the next trial. When A_1_ was equal to 0, the program switched directly to this screen. After 20 trials, a screen indicated that the block had finished, asking participants to press any key to continue to the next block. When the third block finished, the program showed a screen saying that the game was over and displayed the amount that the participant earned, which was calculated as the amount earned in one random trial from the 60 trials played by the subject, plus a €10-base.

### Analyses of Behavioral Data

Behavioral data were analyzed using the R software (R Core Team, 2016). To test if the results were normally distributed, we used the Shapiro-Wilk normality tests (R function shapiro.test). The correlation between the subjects’ behavior in the IG and the DG conditions was obtained using a Pearson correlation. To analyze differences between A_2_s under the IG versus the DG conditions, we used a two-sided Mann-Whitney test (R function wilcox.test), with a confidence interval of 95%. For this, we divided the trials into three categories depending on the amount received by P_2_: when *Â_1_* (i.e., A_1_ normalized as A_1_/T = A_1_/12) was less than 1/3, when *Â_1_* was between 1/3 and 2/3, and when *Â_1_* was greater than 2/3. We then compared the subjects’ behavior in the IG and DG, separately for each of the three categories, also using a 95% confidence interval.

Regression analysis was performed using a Linear Mixed-Effect Model, with error clusterized by subjects (R function lme). We expressed the trustor’s response using the regression model:

A^(t)2=β+0βA^1(t)1+βA^2(t)1IG(t)+βI3G(t)+βE^1(t)1+βE^2(t)1IG(t)+ε

where *Â_2_(t)* is the normalized amount subjects sent to P_1_ (*A_2_/3A*_1_; i.e., the amount P_2_ sent divided the total amount available for this trial) in trial *t*, *Â_1_* the normalized amount P_2_ received (*A*_1_/12), *Ê_1_* the subjects’ first-order expectation (i.e., how much they expected to receive), and IG is a dummy variable that was given the value 1 when P_2_ was partnered with a “human” (IG trials) and 0 when they were partnered with a COM (DG trials), thus providing an interaction analysis reflecting the difference between DG and IG conditions. Given that expectations did not have a significant β-value in our regression analyses, and that this model specification presented the smaller log-likelihood value (see section “Results,” in particular Table 1), for the rest of the analyses we used the model of Equation (1):

**TABLE 1:** Mixed-effect model for the regression of Â_2_ on the variables of interest (see Equation 1 and section “Methods”).

Main experiment				|			Control experiment		

Model 1				Model 2			Model 2		
		
	*coef.*	*p*	*c.i.*	*coef.*	*p*	*c.i.*	*coef.*	*p*	*c.i.*
β_*Intercept*_	0.157	0.001**	[0.065 0.249]	0.162	0.000***	[0.095 0.229]	0.386 0.000***		[0.257 0.516]
β_*IG*_	−0.105	0.093	[−0.228 0.018]	−0.111	0.008**	[−0.192 −0.029]	−0.240 0.02*		[−0.441 −0.038]
β_*Â1*_	0.060	0.217	[−0.035 0.156]	0.060	0.215	[−0.035 0.156]	−0.251 0.124		[−0.369 0.133]
β_*Â1 ×IG*_	0.214	0.002**	[0.081 0.346]	0.214	0.001**	[0.082 0.346]	0.408 0.009**		[0.102 0.714]
β_*Ê1*_	0.009	0.873	[−0.098 0.116]	-	-	-			-
β_*Ê1 ×IG*_	−0.009	0.899	[−0.156 0.137]	-	-	-			-
Log-likelihood			86.39			90.41	98.12		

*Â_1_, amount subjects received; H, a dummy variable that is 0 for DG and 1 for IG. ^∗^,^∗∗^ and ^∗∗∗^ indicate statistical significance at 0.05, 0.01, and 0.001, respectively.*

(1) A^(t)2=β+0βA^1(t)1+βA^2(t)1IG(t)+βI3G(t)

### Analyses of EEG Data

#### EEG Acquisition

EEGs were recorded continuously while participants played the DIG. Recordings were made from an array of 128 silver-chloride active electrodes mounted on an elastic cap, using standard positioning (10-20 system). Reference electrodes were placed on the left and right mastoids (A1/A2). EEG signals were sampled at 1024 Hz, and amplified using an Active-Two amplifier system (Biosemi, Amsterdam, Netherlands). The ground reference consisted of two separate electrodes: Common Mode Sense (CMS) active electrode and a Driven Right Leg (DRL) passive electrode. Electrode sockets were filled with conducting gel to increase signal quality. Electrode offset was kept below 25 μV. An on-line analog low-pass acquisition filter was set at 256 Hz. Data acquisition was made using the Actiview605-Lores software^2^.

#### ERP Analysis

Offline EEG data analysis was performed using EEGLab (Delorme and Makeig, 2004) and LANToolbox, a Matlab toolbox built using algorithms implemented in Fieldtrip, EEGLab, and Cronix, and specifically designed for advanced EEG signal analyses^3^ (see, for example, our previous work: Billeke et al., 2017a,b; Larrain-Valenzuela et al., 2017; Figueroa-Vargas et al., 2020). EEG data for 3 of the 20 participants were excluded because they had more than 40% of trials rejected, based on semiautomatic criteria.

Preprocessing was made by applying a band-pass filter between 0.1 and 100 Hz to the raw signal. Epochs were extracted in the time range between [-1.5 and 1.5] s, centered on the time when subjects were notified about the allocation they received (A_1_). Eye-blinks were identified applying a threshold of 100 μV, and removed using independent-component analysis (ICA) on the signal. Noisy trials were identified by visual inspection and excluded. Signals were filtered using a low pass filter of 40 Hz, and evoked activity was computed as the average signal recorded at each electrode, for all the participants. Baseline was based on the signal recorded [-0.3, -0.05] s. For visualization purposes, a low-pass filter of 20 Hz was applied.

#### Source Estimation

For the estimation of cortical sources, electrode activity (first referenced to mastoids electrodes) was re-referenced to the average of all electrodes. A brain model taken from the anatomy of a standard human brain was used to project scalp activity onto the cortical surface (Montreal Neurology Institute; MNI/Colin27). We defined 5000 cortical sources with 3 orthogonal dipoles each (thus, 3X sources). A three layer conductivity model (brain, skull, and scalp) and a physical forward model (Clerc et al., 2010) was calculated.

Source estimation was computed using an inverse solution based on a weighted minimum norm estimate (wMNE), based on Billeke et al. (2015), as implemented in Brainstorm software (Tadel et al., 2011). Current source density time series for each cortical source was computed with unrestrained dipole orientation, for the average for each condition and for each subject. The activity *x* of *N* electrodes over time (*t*), *X(t)* = *[x_1(t), x_2(t),., x_n(t)]*, was assumed to be linearly correlated with a set *Y* of *M* cortical sources over time *Y(t)* = *[y_1(t), y_2(t),., y_m(t)]* and with additive noise *N(t): X(t)* = *LY(t)* + *N(t)*, where *L* is the physical forward model. An inverse solution was derived as *Y(t)* = *MX(t)* = *RLT(LRLT* + λ *2C) – X(t)*, where *M* is the inverse operator, *R* is the source covariance, *C* the noise covariance, and λ a regulatory parameter, set to 1/3 (Lin et al., 2006). With this, we obtained a time-series of the electrical activity for each cortical source.

#### Statistical Analyses of EEG Data

For ERP analysis, we took the grand average ERP for all subjects for the time epochs when they were notified about the amount A_1_, and grouped them depending on whether they were measured under the IG or the DG conditions. We compared, separately, the results obtained under both conditions using Wilcoxon signed rank test, as implemented in the LAN Toolbox. Signals were projected onto a three-dimensional space, and the adjacent areas with significant differences in this space were corrected using a cluster permutation test (Maris and Oostenveld, 2007). We defined the clusters as groups of adjacent points that showed the same effect, with a threshold of *p* < 0.05. In order to compare the EEG activity obtained in trials when P_2_s received a high versus low A_1_ feedback, we sorted the results depending on whether A_1_ was greater or smaller than €6, then compared the results from both groups for statistical differences, under IG and DG conditions.

### EEG Activity and Behavioral Parameters

To investigate the relationship between a subject’s EEG activity and the correlation between A_2_ and A_1_ (β-value in the regression of Equation 1), we calculated, for each subject and separately for the IG and the DG trials, the average frontomedial activity, composed of activity from electrodes [C12, C13, C14, C19 (AFz), C20, C21 (Fz), C25, C26, C27]. To assess the relationship between β-values and a subject’s frontomedial activity, we specified two separate models (R command *lm*), one for the IG and the other for the DG trials, both of the form:

(2) β(s)=γ+0γF1MA(s)+ε

where FMA(s) was the subject’s average frontomedial activity in the time epoch when they were notified about A_1_, and β is the estimated β-value of the regression of A_2_ on A_1_, calculated for each subject (see Equation 1).

### Control Experiments

In order to replicate the main behavioral results, and to determine whether differences in P_2_’s behavior could respond to differences in endowments of P_1_ between the IG versus the DG conditions, we performed a control experiment, where the payoff structure in DG trials was identical to that in the IG trials, the only difference being that subjects were instructed that the distribution in the DG trials was made by COM, not by P_1_.

#### Participants

Fifty undergraduate students (mean age = 21.3 years; s.d. = 3.7 years; min = 18 years; max = 26 years; 58% women) were recruited both through direct contact and via a mailing list. The control experiments were carried out during a first session at the Computer Labs of the Escuela de Psicología, Universidad de Valparaíso (UV) and during a second session at the Computer Labs of the Escuela de Medicina, Universidad del Desarrollo (UDD), Chile. Participants were paid CLP $5000 (about €6.5) base plus the amount they received in the game. The experimental procedures were the same as those described in the Main Experiment, with the following differences:

#### Instruments

Subjects performed one block of 20 trials of a recursive interactions DIG. Participants were told that they would play with 20 different P_1_s located in a different room of the corresponding facility. Thus, they played once with each P_1_. As in the Main Experiment, all P_1_ and COM allocations were computer simulations, sending a random allocation of tokens drawn from a uniform distribution in [0, 12], with each unit corresponding to CLP $500.

#### DG Trials

In these trials P_1_s received an allocation equal to the amount that COM did not send to P_2_. This meant that the payoff structure in DG trials was identical to that of the IG trials, the only difference being that subjects were instructed that the distribution in the DG trials was made by COM, not by P_1_. This setup served to determine whether differences in the endowments of P_1_ and P_2_ could modify P_2_’s behavior.

#### Experimental Procedure

Participants only performed the behavioral task; no EEG recordings were made. Stimuli were presented using the Psychopy software (Peirce, 2009). The stimuli presentation protocol was the same as that used in the Main Experiment, except that it did not display the screen with the fixation cross.

## Results

**FIGURE 2:**
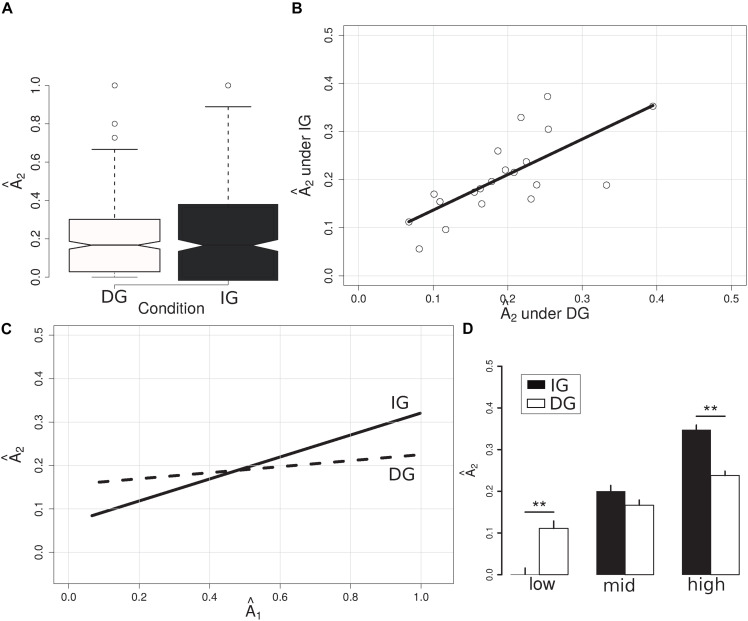
Summary of allocations made under the DG and the IG conditions. **(A)** Barplot of allocations made under DG (white) and IG (black) conditions. No significant differences were found when we considered the allocations made over the entire range of possible endowments. **(B)** Scatterplot of mean individual allocations (open circles) under the DG (*X*-axis) versus the IG (*Y*-axis) conditions; black line corresponds to the Pearson correlation (*r* = 0.72; *p* = 0.0004). Areas of positive and negative reciprocity as a function of the amount received. **(C)** Participants’ normalized allocations as a function of the normalized amount they received (*X*-axis). Lines represent the linear regression of Â_2_ on Â_1_ (see Equation 1) under IG (solid line) and DG (dashed line) conditions. **(D)** Bar plot showing the mean allocations made by subjects (*Y*-axis) under the IG (black bars) and DG (white bars) conditions, for different levels of received trust (*X*-axis); low: Â_1_ < 1/3; mid: 1/3 = < Â_1_ < 2/3; and high: Â_1_ > = 2/3. **indicates *p* < 0.01.

On average, subjects (P_2_s) reciprocated (to P_1_s) an allocation Â_2_ (A_2_/3A_1_; see Methods) of similar magnitude under the DG and IG conditions of the DIG (average for DG: 0.19; median: 0.17; s.d.: 0.17; average for IG: 0.21; median: 0.18; s.d.: 0.19; *p* = 0.39, *W* = 160860; Mann-Whitney test) (Figure 2A). Interestingly, we found a strong correlation between a given subject’s behavior in the DG and their behavior in the IG (*r* = 0.72; *t* = 4.36; *p* = 0.0004; Pearson correlation) (Figure 2B).

To understand how subjects repay trust under outcome-based versus intention-based conditions, we regressed subjects’ Â_2_s on the Ê_1_s and Â_1_s they received under the DG versus the IG conditions of the DIG (Equation 1). Under the DG condition we found that subjects sent the same proportion of their endowment to P_1_, regardless of the amount P_2_s received (β_Â1_ = 0.060; *p* = 0.215; c.i. = [-0.035, 0.156]) (Figure 2C and Table 1). In contrast, under the IG condition we obtained a significant and positive value for β (β_*Â1 ×IG*_ = 0.214; *p* = 0.001; c.i. = [0.082, 0.346]). Thus, this dependence of A_2_ on A_1_ is specific to intention-based behaviors. We did not find significant associations between subjects’ first-order expectations (*Ê_1_*) and behavior, neither for the outcome- nor the intention-based conditions (for instance, β_*Ê1 ×IG*_ = −0.009; *p* = 0.899; c.i. = [−0.156, 0.137]; see Table 1). In addition, the regression model including expectations showed a smaller log-likelihood value as compared to the model of Equation (1) (see Table 1).

We next tested for the existence of regions in which negative and positive reciprocity could be observed. We defined these regions as investment ranges in which subjects playing in the IG condition would send amounts smaller or greater than what they would send under the DG condition, respectively (see section “Methods”). Wilcoxon tests revealed significant differences when Â_1_ was in the [0, 1/3] range (*W* = 6818.5; *p* = 5.16 × 10^–6^; difference in location = −4.17 × 10^–5^) and in the [2/3, 1] range (*W* = 35656, *p* = 1.23 × 10^–8^; difference in location = 0.083), failing to show a difference in location for Â_1_ in the (1/3, 2/3) range (*W* = 18382, *p* = 0.49). Thus, the DIG setup allowed us to unmask three different behaviors: (i) negative reciprocity, where the amount subjects sent was lower in the IG than in the DG; (ii) an area where the behavior of subjects playing IG and DG was indistinguishable; and (iii) positive reciprocity, where the amount subjects sent was higher in the IG than in the DG (see Figures 2C,D).

The results in the control experiments were consistent with our previous findings, namely that the subjects’ allocation, Â_2_, depended on the amount received by P_2_ only under the IG, and not under the DG condition (β_*Â1*_ = −0.251; *p* = 0.124; β_*Â1 ×IG*_ = 0.408; *p* = 0.009; three subjects were excluded from this analysis because they used a strictly self-interested strategy in more than 80% of the trials). In addition, the values for the dummy variable also supports the existence of regions of negative and positive reciprocity (dummy variable for the IG condition: β_*IG*_ = −0.240; *p* = 0.02) (See Table 1).

**FIGURE 3:**
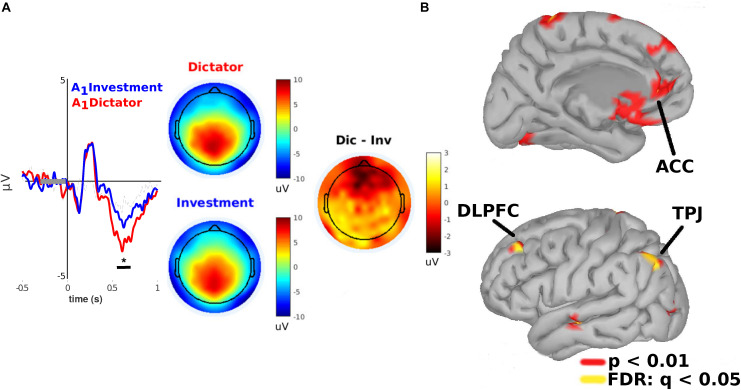
Frontomedial negativity (FMN) is greater under DG condition than under IG condition when subjects are notified about the allocation they will receive (A1). **(A)** *Left*: ERPs amplitude (*Y*-axis) of subjects when they received an allocation from a human (blue lines) versus a COM (red lines). Statistically significant differences occurred between 550 and 680 ms (*X*-axis) after stimulus onset. *Right:* Scalp potentials distribution. **(B)** Cortical source projections (FDR: *q* < 0.05; *p* < 0.01, uncorrected). *indicates *p* < 0.05.

We next analyzed the ERPs of subjects centered on epochs in the range between [−0.5, 1] s relative to when they were notified about the allocation A_1_ they received. We found a significant modulation between 560 and 680 ms after stimulus presentation (*p* < 0.01; cluster-based permutation test; cluster threshold detection: *p* < 0.05; Wilcoxon test paired samples) in a frontomedial ROI of electrodes (*a priori* selection, see section “Methods”). Specifically, subjects displayed a more prominent frontal negativity when they were notified about A_1_ in the DG versus the IG condition (see Figure 3A). Estimations of the cortical sources of these differences projected to the left dorsolateral prefrontal cortex (DLPFC), the left anterior cingulate cortex (ACC), and the left temporoparietal junction (TPJ) (*p* < 0.01; uncorrected; FDR: *q* = 0.05, Figure 3B).

**FIGURE 4:**
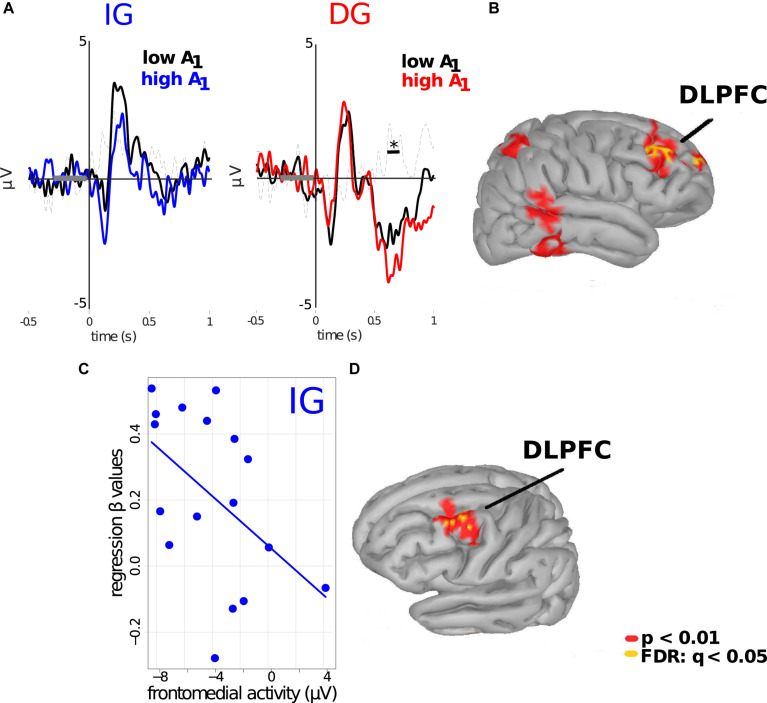
Frontomedial negativity encodes the magnitude of A_1_ under the DG condition. **(A)** *Left:* ERPs of subjects when they received an allocation in the IG. Blue and black lines represent subjects’ ERP when values of A_1_ were above and below €6, respectively. *Right:* ERPs of subjects for the DG condition. Red and black lines represent subjects’ ERP when values of A_1_ were above and below €6, respectively. Significant differences were obtained in a time windows between 550 and 650 ms after stimulus onset, specifically for this condition. **(B)** Cortical source projections (FDR: *q* < 0.05; *p* < 0.01, uncorrected). *indicates *p* < 0.05. Subjects’ average FMN predicts their responses to trust: **(C)** Regression of average ERPs in the frontomedial cluster (*X*-axis) versus the β-values obtained from Equation 1 (*Y*-axis) for the IG (intentions-based) condition (γ*_1_* = -0.037; *p* = 0.045). **(D)** Cortical source projections (FDR: *q* < 0.01; *p* < 0.005, uncorrected).

We next focused specifically on the results obtained under the outcome-based (DG) condition. As the frontomedial negativity was more prominent when subjects were notified about A_1_ in the DG trials, we hypothesized that the magnitude of the received endowment might modulate this potential. For this, we divided the trials depending on whether A_1_ was above or below the median of the range of A_1_ (A_1_ = €6). We found significant differences between conditions (*p* < 0.01; cluster-based permutation test; cluster threshold detection: *p* < 0.05; Wilcoxon test paired samples), with ERPs for trials in which subjects received an A_1_ > €6 being associated with more negative frontomedial activity, as compared to the ERPs for trials in which A_1_ < €6. No such differences were found for IG trials (Figure 4A). Thus, the amplitude of the frontal negativity depended on the magnitude of the amount that subjects received specifically in the outcomes-based conditions. Cortical projections suggest that the right DLPFC was the cortical source of this difference in ERPs (*p* < 0.01; uncorrected; FDR: *q* < 0.05, Figure 4B).

Finally, we focused on the frontomedial negativity that subjects displayed in the IG trials. We explored whether the individual’s mean potentials in the frontomedial ROI between 560 and 680 ms after stimulus onset could be predictive of the subject’s behavior, specifically how subjects responded to an additional unit of trust (*i.e.*, the values of β_*Â1 ×IG*_ in the regression of Â_2_ on Â_1_; see Equation 1 and above). Our analyses revealed a significant correlation between both variables (γ*_1_* = −0.038; *p* = 0.046; see Equation 2), showing that subjects with more negative values in this frontomedial cluster presented greater β-values in the behavioral regression (Figure 4C), thus predicting how subjects responded to trust. We did not observe this association for the DG condition (γ*_1_* = 0.003; *p* = 0.8; see Table 2). Cortical source estimations of the coefficients of this regression also projected to the left DLPFC (*p* < 0.01; uncorrected; FDR: *q* < 0.05, Figure 4D).

**TABLE 2:** Linear model for the regression of subjects’ frontomedial activity on the individuals’ predicted β of the behavioral regression (see Equations 1 and 2).

DG			IG			
	
*Coef.*	*p*	*c.i*	*coef.*	*p*		*c.i*
β*_*FMA*_*	0.003	0.8	[−0.016 0.018]	−0.038	0.046*	[−0.075 −0.012]

*FMA, subjects frontomedial activity. ^∗^indicates statistical significance at 0.05.*

## Discussion

Here we combined electroencephalography and two canonical behavioral economic games to investigate a subject’s behavior and neurophysiological activity in contexts that called for an altruistic response—which requires only concerns about outcome, versus a reciprocal one—which requires concerns about both outcomes and intentions.

We directly compared these two responses by devising a mixed Dictator-Investment game, and contrasting the amount subjects sent in an intention-based condition (the Investments game trials, IG) with the amount that the same subject sent back under an outcome-based condition (the Dictator game trials, DG). We found that subjects displayed other-regarding behaviors, allocating amounts greater than €0 most of the time. In addition, a subject’s behavior depended on which game they were playing. Indeed, we found that only under the intentions-based condition the proportion of the endowment sent increased when the subject received greater allocations, suggesting that the ascription of intention was responsible for the differences in behavior. In addition, we found a strong correlation between a subject’s behavior in both games, also previously reported between the DG and the Prisoner’s dilemma (Capraro et al., 2014). This phenomena is consistent with the findings on cooperative phenotypes (Capraro et al., 2014; Peysakhovich et al., 2014) and moral preferences (Capraro and Rand, 2018).

However our sample size is small (and would be useful to replicate these results with a bigger sample), our results extend those from previous reports, in which subjects’ altruism and reciprocity were compared only for specific values of endowment (for outcomes-based conditions) or received trust (for intentions-based conditions) (*i.e.*, Cox, 2004; Ashraf et al., 2006). By testing how the same subject behaved over a whole range of possible endowments, our results allow us to distinguish three different phenomena: (i) the previously reported positive reciprocity (McCabe et al., 2003; Cox, 2004; Ashraf et al., 2006), for high amount of received trust; (ii) an area where altruism and reciprocity were indistinguishable; and (iii) an area of negative reciprocity, for low levels of trust. Thus, we found that reciprocity is positive only for high amounts of trust, but turns negative (i.e., is less than expected for altruism) for lower amounts.

Positive reciprocity has been discussed elsewhere (McCabe et al., 2003; Cox, 2004; Ashraf et al., 2006). Negative reciprocity could be interpreted as indicating that subjects feel social betrayal if the amount of trust received is less than what they expect from a certain social norm of expected trust (Gouldner, 1960; Coleman, 2000). Similarly, in the Ultimatum Game subjects have been reported to reject an offer (even at a cost to themselves) if they think it is unfair (Güth et al., 1982; Sanfey et al., 2003; Billeke et al., 2013, 2014, 2015; Kaltwasser et al., 2016). Similarly, in the Public good games with altruistic punishment (Fehr and Gächter, 2002), subjects incur a cost to punish free-riders. A crucial difference with the negative reciprocity reported here is that, in our experimental setting, subjects do not lose money when punishing a (perceived) unfair treatment—instead, they earn a greater amount.

At the neurobiological level, a late frontal negativity was more negative when subjects were notified about the amount they had to share in the outcome- versus the intention-based condition. Cortical source estimations for the ERP differences projected to ACC, DLPFC, and TPJ, brain regions which participate in mentalization and cognitive control networks. ACC participates in cognitive control processes in both social (Apps and Ramnani, 2014; Apps et al., 2016) and non-social (Holroyd and Coles, 2002; Shenhav et al., 2013, 2016; Hauser et al., 2014; Ullsperger et al., 2014; Kolling et al., 2016) scenarios. ACC has also been associated with the maintenance of trust and reciprocal interactions (King-Casas et al., 2005; Baumgartner et al., 2008) and its activity is modulated by the “prosocial” neuropeptide oxytocin (Baumgartner et al., 2008; Aspé-Sánchez et al., 2016). In addition, theta activity projecting to ACC might reflect a behavioral heuristic adaptation to the behavior of others (Billeke et al., 2014). TPJ has been shown to be important in the control of selfish impulses (Hutcherson et al., 2015) and the valuation of others’ outcomes (Hutcherson et al., 2015; Hein et al., 2016). During social interactions, TPJ alpha activity correlated with the anticipation of the other’s behavior, and with the use of mentalization in planning future actions (Billeke et al., 2013, 2015; Melloni et al., 2016; Hill et al., 2017; Soto-Icaza et al., 2019). DLPFC, on the other hand, is involved in strategic and normative decision making (Baumgartner et al., 2011; Chang et al., 2011; Yamagishi et al., 2016), and when matching the other player’s expectations about a social interaction (Chang et al., 2011).

We found that under the outcome-based trials this late frontal negativity was more negative when subjects received greater allocations, with scalp activity projecting to the DLPFC. In this respect, two studies have found that a dictator’s unfair offer elicits, in the recipients, a more negative ERP (specifically a feedback-related negativity) than does a fair offer (Wu et al., 2011)—even in the third-person version of the game (*i.e.*, when subjects observe others receiving the allocation; Mothes et al., 2016). In contrast, here subjects processed the allocations received in the outcomes-based condition not as recipients, but as dictators, with greater allocations from the COM implying a greater endowment to choose to share with their human partner. Since greater frontal negativity was observed when subjects received high versus low amounts, it is possible that subjects recruited more cognitive control in order to inhibit the impulse to be selfish—similar to situations in which feedback-related negativity amplitude indicates more cognitive control (Holroyd and Coles, 2002; Ullsperger et al., 2014). Indeed, EEG signals from both ERP and theta activity projecting to DLFC correlate with cognitive control during development (Zamorano et al., 2020). Consistent with this interpretation, the associated activity projected to structures involved in the normative network (specifically, DLPFC), arguably participating in the process of overriding a subjects’ temptations to keep a greater amount for themselves (Baumgartner et al., 2011; Chang et al., 2011; Yamagishi et al., 2016).

There exists extensive evidence involving the MFN in the evaluation of outcomes far from expectations, in both probabilistic and social tasks (i.e., reward prediction error) (Holroyd and Coles, 2002; Potts et al., 2006; Eppinger et al., 2008; Martin and Potts, 2011; Billeke et al., 2013). In addition to reward evaluation, the MFN is involved in processes that influence future decisions and learning (Eppinger et al., 2008; Billeke et al., 2013; Wang et al., 2016; Zhong et al., 2019), being relevant in social tasks where the outcome evaluations are not the final resource allocation of the game. For example, for the same offer evaluation, subjects playing the Ultimatum game show a greater MFN than that they show in the DG (Zhong et al., 2019). In the present experiment, we found a larger MFN for higher offers under the DG condition, which is contrary to the prediction given by reward expectation (Boksem and de Cremer, 2010). However, this result could be interpreted as indicating positive inequity aversion: evidence shows that subjects in the role of proposers display greater MFN evoked by an unfair (but advantageous) outcome distribution than if they have the choice to make a fair (but not advantageous) distribution (Wang et al., 2016).

Interestingly, these effects did not occur under IG. Under this condition, FMN did not show a modulation related to the initial endowment, but was modulated by the prosocial decision that followed. This might occur because reciprocal behavior is more common in nature (Johnson and Mislin, 2011; Franzen and Pointner, 2013): we trust and repay trust from conspecifics everyday, so this outcome requires less cognitive control. An additional—and complementary—interpretation could be that allocations under intentions-based conditions are processed in a more heuristic fashion. Much research has indicated that, in social dilemmas, subjects apply different rules (heuristics) without necessarily recruiting neurophysiological markers of cognitive control (Billeke et al., 2014; Yamagishi et al., 2016; Hill et al., 2017; For a review, see Capraro, 2019). Interestingly, these different rules that subjects apply correlate with activity in medial prefrontal regions, the DLPFC and the TPJ (Billeke et al., 2014; Melloni et al., 2016; San Martín et al., 2016; Yamagishi et al., 2016; Hill et al., 2017). The use of a heuristic strategy in our experiments is supported by the fact that the subject’s average ERP amplitudes showed a significant negative correlation with how much they increased (decreased) their reciprocity when facing more (less) trust. This correlation projects to the DLPFC, which suggests the use of normative rules (Lim et al., 2016). Neurophysiologically, negative reciprocity has previously been investigated only in association with the rejection of unfair offers in the Ultimatum game, in which brain areas related to cognitive control and normative decision-making (such as the DLPFC) correlate with the rejection of unfair offers (Sanfey et al., 2003). Our results suggest that DLPFC activity is associated with the observed behavior in complex social interactions, such as those requiring reciprocity. A final interpretation could be that subjects with more pronounced FMN activity manifest an increase in negative reciprocity. Indeed, evidence shows that FMN activity correlates with the negative reciprocity displayed by the subject in the Ultimatum game, especially for high/fair offers (Kaltwasser et al., 2016).

In summary, the use of the hybrid DIG revealed that reciprocal behavior is positive for high amounts of received trust, but negative for low amounts. In addition, altruism and reciprocity evoke different activity in brain networks involved in mentalizing and cognitive control, which are involved in the inhibition of selfish behavior, and the processing of the internal states, the perspectives, and even the monitoring of the performance of others, such as during (vicarious) reward prediction errors (Amodio and Frith, 2006; Apps et al., 2016; Wittmann et al., 2016). Thus, our findings expand our current knowledge about the relationship between brain networks involved in mentalizing and cognitive control processes, social preferences, and complex social behaviors.

## Data Availability Statement

The datasets generated for this study are available on request to the corresponding author.

## Ethics Statement

The studies involving human participants were reviewed and approved by the Scuola Internazionale Superiore di Studi Avanzati (SISSA) bioethics committee. The patients/participants provided their written informed consent to participate in this study.

## Author Contributions

MA-S, PB, CR-S, and JE conceived and designed the experiments. MA-S and PM performed the behavioral and electrophysiological recordings, and analyzed the data. MA-S wrote the manuscript. PB, CR-S, JE, and RR revised critically the manuscript. All authors contributed to the article and approved the submitted version.

## Conflict of Interest

The authors declare that the research was conducted in the absence of any commercial or financial relationships that could be construed as a potential conflict of interest.

## Footnotes

1 www.neurobs.com

2 www.biosemi.com

3 http://neuroCICS.udd.cl/
